# Sleep problems in children with autism spectrum disorder: a multicenter survey

**DOI:** 10.1186/s12888-021-03405-w

**Published:** 2021-08-16

**Authors:** Hongyu Chen, Ting Yang, Jie Chen, Li Chen, Ying Dai, Jie Zhang, Ling Li, Feiyong Jia, Lijie Wu, Yan Hao, Xiaoyan Ke, Mingji Yi, Qi Hong, Jinjin Chen, Shuanfeng Fang, Yichao Wang, Qi Wang, Chunhua Jin, Tingyu Li

**Affiliations:** 1 Children’s Hospital of Chongqing Medical University, Chongqing Key Laboratory of Childhood Nutrition and Health, National Clinical Research Center for Child Health and Disorders, Ministry of Education Key Laboratory of Child Development and Disorders, Chongqing, 400014 China; 2 Xi’an Children’s Hospital, Xi’an, 710003 China; 3 Department of Children Rehabilitation, Hainan Women and Children’s Medical Center, Haikou, 570100 China; 4 Department of Developmental and Behavioral Pediatric, the First Hospital of Jilin University, Changchun, 130021 China; 5 Department of Children’s and Adolescent Health, Public Health College of Harbin Medical University, Harbin, 150081 China; 6 Department of Pediatrics, Tongji Hospital, Tongji Medical College, Huazhong University of Science and Technology, Wuhan, 430030 China; 7 Child mental health research center of Nanjing Brain Hospital, Nanjing, 210013 China; 8 Department of Child Health Care, the Affiliated Hospital of Qingdao University, Qingdao, 266003 China; 9 Maternal and Child Health Hospital of Baoan, Shenzhen, 518133 China; 10 Department of Child Healthcare, Shanghai Children’s Hospital, Shanghai Jiao Tong University, Shanghai, 200040 China; 11 Children’s Hospital Affiliated to Zhengzhou University, Zhengzhou, 450053 China; 12 NHC Key Laboratory of Birth Defect for Research and Prevention, Hunan Provincial Maternal and Child Health Care Hospital, Changsha, 410008 China; 13 Deyang Maternity & Child Healthcare Hospital, Deyang, 618000 Sichuan China; 14 Department of Children Health Care, Capital Institute of Pediatrics, Beijing, 100020 China

**Keywords:** Sleep problems, ASD, Children, Sleep hygiene, Multicenter

## Abstract

**Background:**

High prevalence of sleep problems have been reported in children with Autism Spectrum Disorder (ASD). This study aims to investigate the sleep conditions of ASD children in China, and explore the relationship between the common sleep problems and core symptoms and developmental levels.

**Methods:**

Using a cross-sectional design, we included 2 to 7-year-old children from 13 cities in China: 1310 with ASD and 1158 with typically-developing (TD) children. The neurodevelopmental level was evaluated with the revised Children Neuropsychological and Behavior Scale (CNBS-R2016). ASD were diagnosed with DSM-5 and Child Autism Rating Scale (CARS). the Social Responsiveness Scale (SRS), the Autism Behavior Checklist (ABC) and the communication warning behavior sub-scale in CNBS-R2016 valued autism behaviors. The children’ s sleep habits questionnaire (CSHQ) assessed sleep conditions.

**Results:**

The prevalence of sleep disorders in ASD children was significantly higher than that in TD (67.4% vs. 51%, *p* < 0.01), and among them the four dimensions with the highest prevalence of sleep problems were bedtime resistance (25.6%), sleep anxiety (22.7%), sleep onset delay (17.9%) and daytime sleepiness (14.7%). ASD children with sleep onset delay or sleep anxiety had higher ABC, SRS scores and higher scores on communication warning behavior with sleep anxiety, with daytime sleepiness had higher ABC, SRS and CARS scores, and with bedtime resistance had higher SRS total scores. Differences in the neurodevelopmental level were not significant.

**Conclusion:**

Children with ASD have a higher prevalence of sleep problems. Bedtime resistance, anxiety, sleep onset delay and daytime sleepiness may be related to the core symptoms, but not be related to the developmental level in ASD children. In the clinic, sleep assessment should be included in the routine of ASD visits, and during the intervention, sleep hygiene education is as important as the treatment of biological factors.

**Trial registration:**

The study was approved by the ethics committee of the Children’s Hospital of Chongqing Medical University, Approval Number: (2018) IRB (STUDY) NO. 121, and registered in the Chinese Clinical Trial Registry (Registration number: ChiCTR2000031194).

**Supplementary Information:**

The online version contains supplementary material available at 10.1186/s12888-021-03405-w.

## Background

Autism Spectrum Disorder (ASD) is a complex neurodevelopmental disorder that seriously endangers human health. Deficits in social communication and repetitive and stereotyped interests and behaviors are its characteristics [1]. The latest report in the United States shows that the prevalence was 1/54 in 2020 [2]. In China, the prevalence has risen from 0.35% in 2018 [3] to 0.7% in 2020 [4]. It is one of the fastest-growing pediatric diseases and has attracted worldwide attention. Furthermore, ASD often accompanies sleep difficulties. Less than 50% [5–8] of typically developing children experience sleep disorders, but 50–80% [9–12] of ASD children. Common sleep problems are trouble falling asleep, decreased sleep duration, sleep onset delay, and night waking [13–15]. Other researchers have found high rates of bedtime resistance and daytime sleepiness [16].

These sleep problems can cause a series of effects. Sleep disorders may adversely affect children’s daily function, affecting behavior, learning, memory regulation and cognition [17–20]. It may also cause emotional problems such as aggression, irritability, over-reactivity and depression [21, 22]. Moreover, disorders also negatively impact ASD symptoms. For example, ASD children with sleep problems showed more severe social skills deficits, and they scored lower on social tests [10, 15, 23, 24]. Other studies have found that core symptoms also affect sleep, such as communication difficulties may exacerbate sleep disorders. All these problems not only reduce the quality of life, but also influence the effect of intervention. These connections all illustrate the severity of sleep problems in ASD. To date, most of these connections have focused on the general concept of “sleep disorders” or “duration of sleep”, and few have linked other dimensions of sleep problems to ASD symptoms. The same problem exists when discussing the relationship between developmental level and sleep disorders. Many children with ASD also suffer from intellectual disability (33% of individuals with ASD have IQ scores of 70 or less), and 24% in the borderline range [2]. Research results on whether developmental level or intelligence affect sleep are also inconsistent [15, 25–29].

Behavioral insomnia in children is considered the most common cause of sleep disorders in children. Also in ASD. So far, behavioral interventions, sleep education, environmental changes and exogenous melatonin are the most effective ways to promote sleep in ASD [30]. The Autism Treatment Network (ATN) proposes to behavioral strategies, parent education about environmental modification, and positive bedtime routines as the first-line approach [31]. However, in China, there are few epidemiological studies on sleep in ASD children. Due to cultural, geographic, and resource constraints, do Chinese children with ASD have more sleep behavior or hygiene problems? Therefore, for the first time, we conducted a large sample, multi-center cross-sectional survey, combined with a case-control study. The main purpose of this study has two, one is to investigate the prevalence of sleep problems and common sleep problems in ASD children in China, the other is to explore the correlation between common sleep problems and ASD core symptoms and developmental levels in Chinese ASD children. Our research helps to understand the sleep conditions of Chinese ASD children and provides a basis for precise intervention in the future.

## Methods

### Patients

#### Participant and selection criteria

This research is part of the China Multi-Center Preschool Autism Project (CMPAP) [32], and collected data on children from 13 cities in China from March 2018 to December 2019. A cross-sectional study was used to investigate sleep problems in children with ASD and TD. It is difficult to conduct stratified random sampling or cluster sampling because of the ability to diagnose and treat autism varies across the country. However, Subspecialty Group of Developmental and Behavioral Pediatrics, the Society of Pediatrics, and the Chinese Medical Association have good diagnosis and treatment capabilities. The cities where these units are located in major regions of China, which can better reflect the sleep conditions of Chinese children. Therefore, we took these cities as centers to recruit children. These13 cities are Harbin, Changchun, Qingdao, Shenzhen, Haikou, Shanghai, Nanjing, Chongqing, Deyang, Xi’an, Wuhan, Changsha and Zhengzhou. The ASD group from special education institutions and outpatient clinics. The diagnosis of ASD was performed independently by a developmental pediatrician and a psychiatrist using the DSM-5 in combination with the childhood autism rating scale (CARS). Children with the following conditions were excluded: (1) with a history of serious congenital diseases; and (2) comorbid other psychiatric disorders. The TD group were recruited from online volunteers. The exclusion criteria were as follows: 1) any serious congenital disease; 2) any developmental or psychiatric disorders, 3) any social developmental or language disorders.

#### Sample size

The sample size calculation used the formula of two independent sample rates:

$$ \mathrm{n}1=\mathrm{n}2=\frac{{\left[{Z}_{a/2}\sqrt{2\kern0.5em \overline{p}\left(1-\overline{p}\right)}+{Z}_{\beta}\sqrt{p_1\left(1-{p}_1\right)+{p}_2\left(1-{p}_2\right)}\right]}^2}{{\left({p}_1-{p}_2\right)}^2} $$

It was reported that the highest prevalence of sleep problems in the ASD was 80%, and in TD was 50%. Set alpha was 0.05, beta was 0.1, and missing rate was 10%. The sample size required for each groups was 57 in each centers through calculation.

All participants’ parents signed an informed consent forms. The study was approved by the ethics committee of the Children’s Hospital of Chongqing Medical University, Approval Number: (2018) IRB (STUDY) NO. 121, and registered in the Chinese Clinical Trial Registry (Registration number: ChiCTR2000031194).

### Scales and questionnaires

#### Demographic questionnaires

Demographic Questionnaires included age, gender, region (North, South, West, East and Middle), residence (urban and rural), parental education level (primary education, secondary education and above), annual family income (< 40,000 RMB, 40000–200,000 RMB and above).

#### The Children’s sleep habits questionnaire (CSHQ)

The Children’s sleep habits questionnaire (CSHQ) is an international standard questionnaire for the assessment of children’s sleep. CSHQ consisted of 33 scoring items and divided into eight dimensions: bedtime resistance, sleep onset delay, sleep duration, sleep anxiety, night waking, parasomnia, sleep disordered breathing and daytime sleepiness. Parents were asked to recall sleep behaviors occurring in a recent week (rarely = sleep behavior occurring 0 to 1 times/week; sometimes = sleep behavior occurring 2 to 4 times/week; usually = sleep behavior occurring 5 to 7 times/week) [33]. Assigned 1, 2, 3 points according to the options. Higher total scores imply more serious sleep problems. A total score ≥ 41 indicates sleep disorder, and the criterion for each dimension is that the score of each dimension exceeded Mean + 2SD. The cut-off score of each dimension are 10.84 (bedtime resistance), 2.31 (sleep onset delay), 5.27 (sleep duration), 7.79 (sleep anxiety), 5.29(night waking), 10.61 (parasomnia), 4.5 (sleep disordered breathing), 15.24 (daytime sleepiness), respectively [34]. CSHQ has been widely used in China, and has good reliability and validity. The. Chinese version of the full scale Cronbach’s alpha coefficient is 0.73; and the sub-scale’s alpha coefficient ranges from 0.42 to 0.69 [35].

### The fifth edition of diagnostic and statistical manual of mental disorders (DSM-5)

DSM-5 is a clinical standard for the diagnosis of ASD, and the diagnosis must meet the following standards: Persistent deficits in social communication and social interaction (Criterion A), and restricted, repetitive patterns of behavior, interests, or activities (Criterion B) currently or by history; These symptoms are present from early childhood and limit or impair everyday functioning (Criteria C and D); These disturbances are not better explained by intellectual disability (Criteria E); according to the intensity of support required, the severity is divided into 3 levels [36].

#### Child autism rating scale (CARS)

CARS is a scale used by professionals to assess the symptoms and severity of autism. There are 15 items in total, and each item is rated as 1 point (behavior equivalent to age), 2 points (mild abnormality), 3 points (moderate abnormality), and 4 points (severe abnormality), respectively. A score between 30 and 36 indicates mild-moderate autism and a score above 36 indicates severe autism [37]. The use of CARS in China has been verified. Cronbach’s alpha coefficient is 0.73 in Chinese version [38].

#### The social responsiveness scale (SRS)

SRS is a scale for parents to fill in the daily social situation of children, concluding 65 items and consisting of 5 sub-scales (social awareness, social cognition, social communication, social motivation and autistic mannerisms) [39]. The score for each item is between 1 and 4. Total

score for typically-developing children should be < 65. The Chinese version of SRS shows that Cronbach’s alpha coefficient is 0.871 ~ 0.922, retest reliability is 0.81 ~ 0.94, and the convergent validity is 0.302 ~ 0.647 [39].

#### The autism behavior checklist (ABC)

ABC is completed by parents to assess autism symptoms. There are 57 items in total, including 5 factors such as sensory stimuli, sensorial relating, body and object use, language and social self-help. The score for each item is between 1 and 4. Scores for children without autism should be < 53 [40]. ABC scale has been widely used in China and the positive coprevalence rate of clinical diagnosis and ABC scale is more than 80% [38].

#### The revised children neuropsychological and behavior scale (CNBS-R2016)

CNBS-R2016 can evaluate the neurodevelopmental levels and autism symptoms of children, including six domains (gross motor, fine motor, adaptive behavior, language, personal society, and autism warning behavior). The developmental quotient less than 70 indicates a developmental delay and communication warning behavior quotient more than 30 is suspected of being at risk of autism [41]. The CNBS-R2016 and the Griffiths Mental Development Scales for China showed good consistency in the developmental assessment of children with ASD [41].

### Statistical analysis

SPSS statistical software 25.0 was used for statistical analysis. Shapiro-Wilk test, histogram, and QQ graph were combined to detect data normality. Continuous variables were described as mean ± standard deviation (M ± SD), medians (inter-quartile ranges) (M (IQR)) and medians (5th percentile to 95th percentile) (P5-P95). Categorical variables were described as frequencies and percentages. Two-sample independent T-test,the chi-square test and Mann-Whitney test were used for comparisons between groups. Logistic regression was used to examine the differences in sleep problems between ASD and TD groups. Significance was presumed at *p* < 0.05.

## Results

### Demographic characteristics

**Fig. 1:**
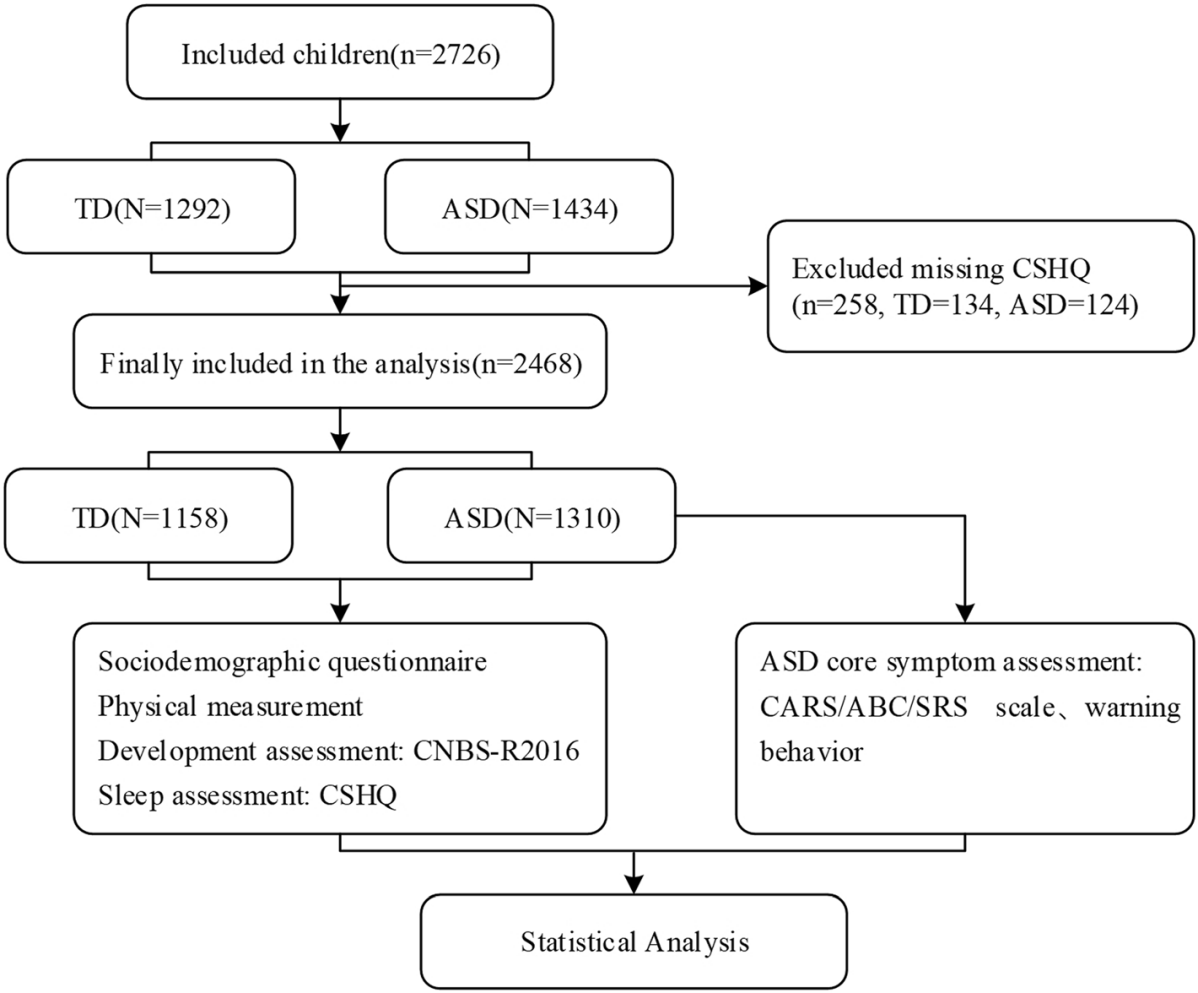
Investigation flowchart

The CMPAP collected 2968 (ASD = 1469, TD = 1499) children. According to the inclusion and exclusion criteria of our study. A total of 2726 children aged 2–7 years were enrolled in the study, and 2468 children were included eventually after excluding 258 children who had a sleep questionnaire loss (Fig. 1). The demographic characteristics of the children are shown in Table 1. This study enrolled 1158 children with ASD (1077 males and 233 females), with a median (IQR) age of 3.95(3.13–4.88). There were 1310 children (765 males and 393 females) in TD group, with a median (IQR) age of 4.44(3.42–5.39). There were statistically significant differences in age and gender distribution between the two groups (*P* < 0.05). Differences were also significant in region, residence, parental education level and annual family income (*P* < 0.05, respectively). Logistic regression can be used to adjust for confounding factors. Therefore, we used it to compare the differences between the two groups. In Model I (unadjusted), the occurrence of sleep disorders was taken as the dependent variable and grouping (ASD and TD) as the independent variable (OR = 1.984, *p* < 0.001). Model II included adjusted factors such as gender, age, region, residence, parents’ educational level and annual family income as independent variables, and the results showed that the differences in sleep problems between the two groups remained (OR = 1.873, p < 0.001) (Additional file 1).

**Table 1:** Demographic characteristics of the participants in TD and ASD groups

Variable	TD(*N* = 1158), Median (IQR)/N(%)	ASD(*N* = 1310), Median (IQR)/N(%)	*Z*/χ2	*P*
Age, years	4.44 (3.42–5.39)	3.95 (3.13–4.88)	− 6.667	<0.001
Gender, N (%)				
Female	393 (33.9)	233 (17.8)	84.701	<0.001
Male	765 (66.1)	1077 (82.2)		
Region				
North	422 (36.4)	376 (28.7)	34.978	<0.001
South	234 (20.2)	291 (22.2)		
West	190 (16.4)	319 (24.4)		
East	158 (13.6)	144 (11)		
Middle	154 (13.3)	180 (13.7)		
Residence				
Urban	1020 (88.1)	893 (68.2)	143.721	<0.001
Rural	108 (9.3)	248 (21.7)		
Miss	30 (2.6)	133 (10.2)		
Paternal education level				
Primary education	7 (0.6)	22 (1.7)	45.714	<0.001
Secondary education	323 (27.9)	516 (39.4)		
Secondary education above	812 (70.1)	752 (57.4)		
Miss	16 (1.4)	20 (1.5)		
Maternal education level				
Primary education	14 (1.2)	33 (2.5)	106.887	<0.001
Secondary education	294 (25.4)	579 (44.2)		
Secondary education above	840 (72.5)	688 (52.5)		
Miss	10 (0.9)	10 (0.8)		
Annual family income, RMB^a^				
< 40,000	380 (32.8)	726 (55.4)	152.34	<0.001
40,000–200,000	490 (42.3)	439 (33.5)		
> 200,000	207 (17.9)	92 (7)		
Miss	81 (7)	53 (4)		

^a^1 RMB ≈ 0.155 US Dollars

In Additional file 2**,** we compared the demographic data of the lost sample with the included sample. The results showed that there were no differences in gender and age, but there were differences in other areas. Then we found there was a large amount of missing demographic data in the lost group, which may be an important reason for the difference in baseline. For example, in the loss group, the missing rate in multiple demographic data was more than 40%, while the included group was 0.8–6.6%. In the included samples, the results of our adjustment of demographic data also showed that they were not confounded with sleep problems. Therefore, the finally included sample can be representative of the whole to some extent.

### Comparison of sleep symptoms between the TD and the ASD group

As shown in Table 2, among the 1310 ASD children, 883 (67.4%) had sleep disorders (CSHQ ≥41), which was significantly higher than that of the control children (51%) (*P* < 0.001), and the total score of CSHQ was also higher (43(36–58) VS. 41(36–51), *P* < 0.001). The prevalence and scores of eight dimensions of sleep problems in ASD children was higher than that in TD: bedtime resistance (25.6% VS. 15.9%, *P* < 0.001, 9(8–14) VS. 9(7–13), *P* < 0.001), sleep onset delay (17.9% VS. 12.8%, *P* < 0.001, 2(1–3) VS. 1(1–3), *P* < 0.001), sleep duration (9.4% VS. 6%, *P* < 0.001, 3(3–7) VS. 3(3–6), *P* < 0.001), sleep anxiety (22.7% VS. 15.7%, *P* < 0.001, 6(5–9) VS. 6(5–8), *P* < 0.01), night waking (5% VS. 1.5%, *P* < 0.001, 3(3–5.45) VS. 3(3–5), *P* < 0.001), parasomnias (7.4% VS. 4%, *P* < 0.001, 8(7–12) VS. 8(7–10), *P* > 0.05), sleep disordered breathing (5.3% VS. 1.3%, *P* < 0.001, 3(3–5) VS. 3(3–4), *P* < 0.001), daytime sleepiness (14.7% VS. 6%, *P* < 0.001, 11(8–19) VS. 11(8–17), *P* < 0.001).

**Table 2:** Comparison of Sleep symptoms between the TD and the ASD group

Variable	Prevalence, N (%)		*χ*^*2*^	*p*	The Score of CSHQ, Mean ± SD/ Median(P5-P95)		*Z*	*p*
	TD (1158)	ASD (1310)			TD (1158)	ASD (1310)		
Bedtime Resistance	184 (15.9)	336 (25.6)	35.203	<0.001	9 (7–13)	9 (8–14)	−6.731	<0.001
Sleep Onset Delay	148 (12.8)	235 (17.9)	12.475	<0.001	1 (1–3)	2 (1–3)	−7.655	<0.001
Sleep Duration	69 (6)	123 (9.4)	10.084	<0.001	3 (3–6)	3 (3–7)	−6.267	<0.001
Sleep Anxiety	182 (15.7)	298 (22.7)	19.397	<0.001	6 (5–8)	6 (5–9)	−3.288	0.001
Night Waking	17 (1.5)	65 (5)	23.358	<0.001	3 (3–5)	3 (3–5.45)	−3.879	<0.001
Parasomnia	46 (4)	97 (7.4)	13.265	<0.001	8 (7–10)	8 (7–12)	−0.349	0.727
Sleep Disordered Breathing	15 (1.3)	69 (5.3)	29.493	<0.001	3 (3–4)	3 (3–5)	−3.762	<0.001
Daytime Sleepiness	69 (6)	193 (14.7)	49.87	<0.001	10 (8–17)	11 (8–19)	−5.16	<0.001
A total score of CSHQ	591 (51)	883 (67.4)	68.462	<0.001	41 (36–51)	43 (36–58)	−9.688	<0.001

Among children with ASD, the four dimensions with the highest prevalence of sleep problems were bedtime resistance (25.6%), sleep anxiety (22.7%), sleep onset delay (17.9%) and daytime sleepiness (14.7%), respectively. The same was true for TD, with prevalence of 15.9, 15.7, 12.8 and 6%, respectively. This was consistent with previous research. However, the prevalence of night waking in ASD is the lowest in our study. Most previous reports have shown that night waking is also a significant sleep problem in ASD.

### Comparison of ABC, SRS, CARS and communication warning behavior scores among ASD children with or without bedtime resistance, sleep anxiety, sleep onset delay and daytime sleepiness

We selected the four most significant sleep problems in the results of this study as common sleep problems of ASD to explore their relationship with core symptoms and developmental levels. Compared with the ASD children without bedtime resistance, ASD with bedtime resistance had higher scores in SRS (*P* < 0.05), but lower scores in the ABC language sub-scale (*P* < 0.05) and CARS *(P* < 0.01). ASD children with sleep anxiety or sleep onset delay had higher ABC scores (*P* < 0.01) and SRS scores (*P* < 0.001), and ASD children with sleep anxiety had higher communication warning behavior scores (*p* < 0.05); there were no significant differences in CARS scores. ASD children with daytime sleepiness had higher ABC scores (*P* < 0.001), SRS scores (*P* < 0.001), CARS scores (*P* < 0.05). (Table 3) Further gender-stratified analysis showed that ASD children with common sleep problems exhibited higher ASD symptom scores both in male and female. But some results were not statistically significant in female (Additional file 3**and** Additional file 4).

**Table 3:** Differences in autism symptoms in ASD children with and without common sleep problems

Item	Bedtime Resistance,Mean ± SD/ Median (IQR)		*Z/t*	*P*	Sleep Anxiety, Mean ± SD/ Median (IQR)		*Z/t*	*P*	Sleep Onset Delay,Mean ± SD/ Median (IQR)		*Z/t*	*P*	Daytime sleepiness, Mean ± SD/ Median (IQR)		*Z/t*	*P*
	(−)	(+)			(−)	(+)			(−)	(+)			(−)	(+)		
ABC																
Sensory stimuli	7 (3–11)	8 (3–12)	−1.632	0.103	7 (3–11)	9 (4–12)	−3.541	<0.001	7 (3–11)	8 (4.75–13)	−3.551	<0.001	7 (3–11)	8.5 (5.25–13)	−3.868	<0.001
Sensorial relating	12.97 ± 7.76	12.74 ± 7.77	0.446	0.655	12.59 ± 7.7	13.89 ± 7.88	−2.425	0.015	12.41 ± 7.67	15.23 ± 7.76	−4.726	<0.001	12.5 ± 7.78	15.13 ± 7.26	−4.162	<0.001
Body and object use	7 (3–12)	7 (3–12)	−0.858	0.391	6 (2–12)	8 (4–13)	−3.316	0.001	6 (3–12)	9 (3–14)	−4.206	<0.001	7 (3–12)	7 (3–13)	−1.328	0.184
Language	11 (6–17)	10 (5–15)	−2.553	0.011	11 (5–16)	11 (5–18)	−1.302	0.193	11 (5–16)	12 (6–19)	−2.166	0.030	11 (5–16)	13 (6–19)	−2	0.046
Social self-help	10.74 ± 5.23	10.56 ± 5.15	0.52	0.603	10.46 ± 5.19	11.42 ± 5.18	−2.675	0.008	10.44 ± 5.21	11.84 ± 5	−3.472	0.001	10.51 ± 5.23	11.69 ± 4.93	−2.793	0.005
Total score	49 (35–66)	49 (33–66)	−0.377	0.706	47.5 (34–64)	54 (38–71)	−3.455	0.001	46 (33–65)	57 (42–75)	−5.453	<0.001	49.74 ± 23.56	56.92 ± 24.31	−3.842	<0.001
SRS																
Social awareness	11.24 ± 3.31	11.9 ± 3.47	−2.836	0.005	11.18 ± 3.28	12.15 ± 3.52	−4.083	<0.001	11.14 ± 3.32	12.63 ± 3.27	−5.812	<0.001	11.19 ± 3.31	12.49 ± 3.4	−4.817	<0.001
Social cognition	17.85 ± 4.83	18.56 ± 4.26	−2.317	0.021	17.68 ± 4.71	19.19 ± 4.53	−4.570	<0.001	17.82 ± 4.71	18.99 ± 4.57	−3.216	0.001	17.78 ± 4.75	19.33 ± 4.26	−4.112	<0.001
Social communication	31.92 ± 9.15	33.73 ± 9.2	−2.838	0.005	31.68 ± 9.1	34.68 ± 9.12	−4.647	<0.001	31.79 ± 9.14	35 ± 8.96	−4.560	<0.001	31.78 ± 9.22	35.41 ± 8.41	−4.949	<0.001
Social motivation	15 (11–19)	15 (11–19)	−0.748	0.454	14 (11–18)	16 (12–20)	−3.495	<0.001	14.92 ± 5.12	16.09 ± 5.04	−2.976	0.003	14.86 ± 5.09	16.52 ± 5.09	−4.041	<0.001
Autistic mannerisms	13 (9–17)	13 (9–18)	−0.3	0.764	12 (8.5–17)	14 (10–19)	−4.548	<0.001	13 (9–17)	14.5 (10–19)	−3.888	<0.001	12 (9–17)	15 (11–19)	−4.55	<0.001
Total score	89.35 ± 23.74	92.95 ± 23.39	−2.198	0.028	88.22 ± 23.18	97.1 ± 24.22	−5.369	<0.001	88.61 ± 23.59	97.72 ± 22.79	−5.014	<0.001	88.57 ± 23.6	98.95 ± 22.31	−5.488	<0.001
CARS																
Total score	33 (28–38)	31 (27–36)	−3.084	0.002	32.91 ± 7.04	33.21 ± 7.07	−0.589	0.556	32.5 (28–37)	33 (29–38)	−1.719	0.086	32.79 ± 6.94	34.25 ± 7.64	−2.322	0.02
Communication warning behavior																
Total score	42.34 ± 22.36	42.61 ± 21.08	−0.164	0.869	41 (24–58)	46 (32–61)	−2.386	0.017	42 (24–58)	45.5 (28.5–62.75)	−1.834	0.067	42 (24–58)	45.5 (31–63.25)	−1.714	0.087

### Comparison of the developmental levels among ASD children with or without bedtime resistance, sleep anxiety, sleep onset delay and daytime sleepiness

It can be seen from Table 4 that there were no differences in developmental quotient among ASD children with or without bedtime resistance, sleep anxiety, sleep onset delay and daytime sleepiness. According to the results of gender stratification, there is still no difference in development quotient between the groups. (Additional files 5 and 6)

**Table 4:** Differences in developmental quotient in ASD children with and without common sleep problems

Item	Bedtime Resistance,Mean ± SD/ Median (IQR)		*Z/t*	*P*	Sleep Anxiety, Mean ± SD/ Median (IQR)		*Z/t*	*P*	Sleep Onset Delay,Mean ± SD/ Median (IQR)		*Z/t*	*P*	Daytime sleepiness, Mean ± SD/ Median (IQR)		*Z/t*	*P*
	(−)	(+)			(−)	(+)			(−)	(+)			(−)	(+)		
CNBS-R2016																
Gross motor	78.42 ± 21.47	78.28 ± 21.05	0.081	0.935	79 (64–92)	79.5 (64–93)	−0.512	0.609	78 (63–91.75)	81 (69.25–93)	− 1.452	0.146	78 (64–91)	81.5 (64.75–94)	− 1.549	0.121
Fine motor	59 (46–73)	59 (47–74)	0.000	1.000	59 (46–73)	59 (48.75–74.25)	−0.799	0.424	59 (46–74)	59 (49–73)	−0.233	0.816	59 (46–74)	60 (46.75–72.25)	−0.211	0.833
Adaptive behavior	62 (49–76)	63 (47–75)	−0.329	0.742	63 (48–76)	62 (48.75–76)	−0.025	0.980	62 (48–76)	65 (50–75.75)	−1.056	0.291	62 (48–75)	65 (49.75–81.25)	−1.657	0.098
Language	49 (34–72)	48 (32–72)	−1.022	0.307	48.5 (33–72)	50 (35–72)	− 0.365	0.715	49 (33–73)	47 (35–63)	−1.114	0.265	48.5 (33–71)	51 (35.75–74)	−0.978	0.328
Personal-social	54 (42–68)	52 (41–66)	−1.372	0.170	53 (42–68)	54 (43–65)	−0.487	0.627	54 (42–68)	51 (43–67)	−0.778	0.437	53 (41.75–68)	54 (44–70.25)	−1.056	0.291
GQ	61 (50–75)	61 (50–72)	−0.609	0.542	61 (50–74.25)	61.5 (51–72.25)	−0.235	0.814	61 (50–74.75)	61 (51–72.75)	−0.101	0.919	61 (50–74)	63.5 (52–78)	−1.403	0.16

## Discussion

At present, this is the first large-scale study on sleep conditions in Chinese ASD children. The 13 cooperative centers have the ability to standardize the diagnosis and treatment of ASD, which can better reflect the sleep conditions of children with ASD. Primarily, the prevalence of sleep difficulties in ASD was 67.4%, which is consistent with previous reports, significantly higher than that in TD (51%), and the scores and prevalence of sleep problems in all dimensions were also higher. These emphasized the severity of sleep problems in ASD. Further analysis found that the first four were bedtime resistance (25.6%), anxiety (22.7%), sleep onset delay (17.9%) and daytime sleepiness (14.7%). Except for these four dimensions, in previous reports, night waking is also a common sleep problem reported by parents, but it was the lowest in our study (5%). This sleep pattern is similar to a recent survey in China and India. China investigated the sleep conditions of 475 preschool children with ASD and found that the prevalence of sleep problems was 81.7%. The four with the highest were sleep resistance (90.9%), sleep anxiety (91.7%), daytime sleepiness (60.7%) and sleep onset delay (59.1%), and the lowest two were night waking (25.4%) and Sleep disordered breathing (19.8%) [42]. In India, the prevalence in 2 to 6 years old children with ASD was 93%. The four with the highest prevalence were sleep resistance (95%), sleep anxiety (85%), sleep duration (81%) and sleep onset delay (66%), the lowest two were night awakening (50%) and daytime sleepiness (27%) [43]. Although they have similar sleep patterns to ours, the prevalence is significantly different. By comparison, these studys defined these types of sleep problems as a sub-scale score above the respective cut off, but did not indicate the cut off value clearly. We used M ± 2SD as the cutoff value, which has been used in other articles. Combined with domestic and foreign research, we found that ASD has a higher detection rate of sleep problems, but the results of specific dimensions are different. This may be related to the survey environment, scale, living habits, survey methods, etc. In fact, there is no consensus on the definition and classification of sleep difficulties in the world and few studies have calculated the prevalence of each dimension in CSHQ, so it is not convenient for us to compare each dimension here. But in general, the most common sleep problems are all difficulties in falling asleep, shortened sleep time, delayed sleep onset and awakening at night. It reminds us that we should pay more attention to these problems when discussing sleep conditions of Chinese ASD children, and we can also explore the sleep patterns of ASD more specifically and comprehensively in future studies.

There are currently a large number of studies on the overall sleep status and autism symptoms of ASD, so we do not separately analyze and discuss it, but focus the research on different dimensions. For this study, it is the four dimensions of with the highest prevalence [44–46].

The sleep problem with the highest prevalence of ASD in this survey was bedtime resistance (25.6%). Children with it had higher SRS scores, which were mainly manifested in social awareness, cognition and communication. Hollway [47] analyzed data of 1583 ASD children and found that social interaction deficits were related to bedtime resistance, and Asians had a higher risk of sleep resistance than other races. Our results echo it. At present, behavioral insomnia of childhood is recognized as the most common cause of sleep disorders in children. It may be related to poor sleep training or environmental restrictions imposed by parents or caregivers, including sleep onset association type (children’s specific dependence on stimuli, people, objects, or settings to start or return to sleep) and limit setting type (behavior of delay or refusal before going to bed due to the difficulty of setting the limit by the caregiver). Some limit setting insomnia of childhood can be manifested as resistance at bedtime [48–51], and a recent study found that sleep hygiene was positively correlated with the resistance [52]. Therefore, due to the behavioral characteristics of ASD children and cultural differences, ASD children in China and even Asia have more sleep hygiene or sleep behavior problems, which leads to a high prevalence of sleep resistance. For example, co-sleeping is common in many Asian countries [43, 53, 54]. Although children had lower ABC language and CARS scores when there was bedtime resistance, there was a trend of higher scores in other sub-scales. CARS are used to measure ASD severity which is more related to biological mechanisms, while bedtime resistance is more likely to be caused by sleep behavior and sleep hygiene. It may be the reason for the opposite trend here of CARS. Anyway, their relationship needs further study.

In this study, ASD children with sleep onset delay and sleep anxiety had higher ABC, SRS and autism warning behavior scores, which were specifically manifested in higher social interaction, communication, cognition, language and stereotyped behavior scores. Tudor [55] found that autism symptoms and severity were associated with short sleep duration and sleep onset delay. Communication symptoms can be predicted by sleep anxiety, and autism severity, stereotyped behaviors and social interaction deficits can be predicted by sleep onset delay. Hollway’s [47] research showed that sleep anxiety was related to abnormal taste and smell perception. Our research further found that these two may be related to social interaction, communication, cognition, language and stereotyped behavior. Special attention should be paid to the sleep problems caused by language disability. Children with autism are often unable to express some demands or discomfort due to their language barriers, which may indirectly aggravate their sleep problems. By this, for ASD children with language impairment, we must pay more attention to their sleep. Earlier we mentioned that there is no consensus on the definition of many sleep problems in the world, one of which is sleep anxiety. Sleep anxiety in CSHQ including afraid of sleeping in dark, afraid of sleeping alone, trouble sleeping away and needs parents in the room to sleep. These cannot fully reflect the anxiety state of children when they fall asleep. A survey of ATN that included 1784 from childhood to adolescence ASD showed that with age increase, the prevalence of sub-items related to sleep anxiety in CSHQ showed a downward trend [56]. Therefore, in future research, we need to figure out whether children have sleep anxiety and may need to observe it by age.

The daytime state is closely related to the study and function during the day. Our research found that daytime sleepiness was related to the core symptoms of autism and children showed higher scores in ABC, SRS, and CARS. A recent study in India found that the severity of autism was positively correlated with daytime sleepiness in male children between 2 and 6 years old [43]. Another study found that daytime sleepiness in toddlers was related to autism characteristics. Toddlers with autism characteristics had a higher prevalence of daytime sleepiness than those without autism characteristics [57]. Hodge [58] reported that the typical developing children of 3–17 years old had a significant decrease in daytime sleepiness with age, but the daytime sleepiness of ASD children still increased. Therefore, daytime sleepiness may be a symptom that can detect children’s ASD early, and we should pay attention to children’s daytime sleepiness. In addition to poor sleep behavior and sleep hygiene, sleep disturbance involves more complex neurobiological mechanisms, especially the abnormal melatonin level. Compared with normal children, ASD had significantly lower levels of melatonin and metabolites, and there were decreased at night, increased during the day, and delayed circadian rhythm [59–65]. This partly explains their delayed onset of sleep, night waking and daytime sleepiness. The underlying biological rhythm and behavioral characteristics of ASD may make them more vulnerable to sleep disorders.

In addition, Gender-stratified analysis showed that ASD children with common sleep problems exhibited higher ASD symptom scores both in male and female, but some results were not statistically significant in female. This may be caused by the small sample size of the girls group. The analysis found that the sample size of boys was 1077 and that of girls was 233.

These four types of sleep problems were found to be related to core symptoms, but not related to the neurodevelopmental level. Previous studies on the relationship between developmental level and core symptoms are inconsistent. Several studies reported that the neurodevelopmental level or intelligence may affect sleep [26, 27, 29]. One research found that intelligence has an effect on sleep anxiety to a certain extent, but has no effect on bedtime resistance and sleep duration [47]. Another study found that intelligence only affects night waking [66]. In contrast, sleep disorders can occur at all neurodevelopmental levels in most studies [15, 25, 27, 28]. The reason for the inconsistency of these results may be that most sleep assessments are based on subjective reports, lack of objective sleep data support, and there are certain differences in the age of children included in different studies. More research is needed to clarify the relationship between sleep disorders and developmental trajectories.

In addition to helping to understand the sleep conditions of Chinese children with ASD, our research also provides directions for precise intervention in the future. Bedtime resistance was a sleep problem closely related to sleep behavior and sleep hygiene, and the most prominent sleep problem in this survey. Daytime sleepiness and sleep onset delay were likely to be related to abnormal melatonin levels. This suggests that in the future intervention, the cultivation of sleep hygiene and behavior is as important as the intervention of biological factors, especially starting from the actual situation of our country, combining our country’s culture and resources to formulate intervention strategies. Moreover, there is still a clinical phenomenon that children with ASD often come to the doctor because of language delay. Parents generally pay more attention to the core symptoms of the child, thus ignoring the co-occurring sleep problems which are related to core symptoms. Therefore, we suggest that in the future diagnosis and treatment, sleep assessment should be routinely included to achieve early prevention, detection and intervention of sleep disorders in ASD children.

This study has limitations. First, Children are recruited online rather than randomly sampled, which may make the sample less representative, and it was designed as a cross-sectional study when comes to the relationship between sleep disorders and core symptoms, which could not establish a causal relationship. Moreover, there was no objective measure of sleep. The CSHQ was completed by parents. This could led to a response bias. So there may be deviations between the real sleep situation and the survey results. It is better to combine subjective and objective assessments when possible. Although the scale assessment was completed by trained professionals, this study involved 13 cities in China, and the quality control was not mature enough, which may also be the reason for the contrary results in the analysis of sleep resistance. Therefore, further objective research is needed.

## Conclusion

The prevalence of sleep problems in ASD is significantly higher than that in typically-developing children at all levels in China. The first four are bedtime resistance, anxiety, sleep onset delay and daytime sleepiness. These sleep problems may be not related to the developmental level with ASD, but be related to their core symptoms. In the clinic, sleep assessment should be included in the routine of ASD visits, and during the intervention, sleep hygiene education is as important as the treatment of biological factors.

## Supplementary Information

**Additional file 1: Table S1.** Difference in the prevalence of sleep disorder between ASD and TD children after adjusting confounders by logistic regression.

**Additional file 2: Table S2.** Comparison of demographic characteristics between lost samples and included samples.

**Additional file 3: Table S3.** Differences in autism syptoms in ASD boys* with and without common sleep problems.

**Additional file 4: Table S4.** Differences in autism syptoms in ASD girls* with and without common sleep problems.

**Additional file 5: Table S5.** Differences in developmental quotient in ASD boys with and without common sleep problems.

**Additional file 6: Table S6.** Differences in developmental quotient in ASD girls with and without common sleep problems.

## Data Availability

The datasets used during the current study are available from the corresponding author on reasonable request.

## Abbreviations

ABC: Autism Behavior Checklist
ASD: Autism Spectrum Disorder
ATN: Autism Treatment Network
CARS: Child Autism Rating Scale
CMPAP: China Multi-Center Preschool Autism Project
CNBS-R2016: Revised Children Neuropsychological and Behavior Scale
CSHQ: Children’ s Sleep Habits Questionnaire
DSM-5: *Diagnostic and Statistical Manual of Mental Disorders,* fifth edition
IQR: inter-quartile range
M ± SD: Mean ± Standard Deviation
*P5-P95*: 5th percentile to 95th percentile
SRS: Social Responsiveness Scale
TD: Typically-developing
