# Supplementary material for: Sleep problems in children with autism spectrum disorder: a multicenter survey
Source: BMC Psychiatry. 2021 Aug 16;21:406. doi: 10.1186/s12888-021-03405-w (PMC8365936; doi:10.1186/s12888-021-03405-w)
Supplement: Supplementary file 1 — Additional file 1: Table S1. Difference in the prevalence of sleep disorder between ASD and TD children after adjusting confounders by logistic regression. [file 12888_2021_3405_MOESM1_ESM.docx]

| Table S1. Comparison of sleep problem between TD and ASD before and after adjustment | | | | |
| --- | --- | --- | --- | --- |
| Model | OR | | 95%CI | *P* |
|  | TD | ASD |  |  |
| Unadjusteda | reference | 1.984 | 1.685-2.336 | ＜0.001 |
| Adjusted modelb | reference | 1.873 | 1.567-2.239 | ＜0.001 |
| a: no adjustment b: adjusted for age,gender,region,residence,parents' educational level and annual family income | | | | |
