# Supplementary material for: Sleep problems in children with autism spectrum disorder: a multicenter survey
Source: BMC Psychiatry. 2021 Aug 16;21:406. doi: 10.1186/s12888-021-03405-w (PMC8365936; doi:10.1186/s12888-021-03405-w)
Supplement: Supplementary file 2 — Additional file 2: Table S2. Comparison of demographic characteristics between lost samples and included samples. [file 12888_2021_3405_MOESM2_ESM.docx]

| **Table S2.** Comparison of demographic characteristics between lost samples and included samples | | | | |
| --- | --- | --- | --- | --- |
| Variable | Included (N=2468), Median(IQR)/N(%) | Lost(N=258), Median(IQR)/N(%) | P |  |
| Age,years | 4.1342（3.25-5.14） | 4.1822（3.32-5.09） | 0.558 |  |
| Gender, N (%) |  |  | 0.98 |  |
| Male | 1842(74.6) | 192(74.7) |  |  |
| Female | 626(25.4) | 65(25.3) |  |  |
| Region |  |  | ＜0.001 |  |
| North | 798(32.3) | 62(24) |  |  |
| South | 525(21.3) | 75(29.1) |  |  |
| West | 509(20.6) | 47(18.2) |  |  |
| East | 302(12.2) | 52(20.2) |  |  |
| Middle | 334(13.5) | 22(8.5) |  |  |
| Residence |  |  | ＜0.001 |  |
| Urban | 1913(77.5) | 112(43.4) |  |  |
| Rural | 392(15.9) | 34(13.2) |  |  |
| Miss | 163(6.6) | 112(43.4) |  |  |
| Paternal education level |  |  | ＜0.001 |  |
| Primary education | 29(1.2) | 1(0.4) |  |  |
| Secondary education | 838(34) | 69(26.7) |  |  |
| Secondary education above | 1563(63.4) | 75(29.1) |  |  |
| Miss | 36(1.5) | 113(43.8) |  |  |
| Maternal education level |  |  | ＜0.001 |  |
| Primary education | 47(1.9) | 2(0.8) |  |  |
| Secondary education | 872(35.4) | 78(30.2) |  |  |
| Secondary education above | 1527(61.9) | 67(26) |  |  |
| Miss | 20(0.8) | 111(43) |  |  |
| Annual family income,RMB* |  |  | ＜0.001 |  |
| <40000 | 1105(44.8) | 74(28.7) |  |  |
| 40000-200000 | 928(37.6) | 26(10.1) |  |  |
| >200000 | 299(12.1) | 9(3.5) |  |  |
| Miss | 134(5.4) | 149(57.8) |  |  |
| *1 RMB≈0.155 US Dollars |  |  |  |  |
