# Supplementary material for: Sleep problems in children with autism spectrum disorder: a multicenter survey
Source: BMC Psychiatry. 2021 Aug 16;21:406. doi: 10.1186/s12888-021-03405-w (PMC8365936; doi:10.1186/s12888-021-03405-w)
Supplement: Supplementary file 3 — Additional file 3: Table S3. Differences in autism syptoms in ASD boys* with and without common sleep problems. [file 12888_2021_3405_MOESM3_ESM.docx]

| **Table S3.** Differences in autism syptoms in ASD boys* with and without common sleep problems | | | | | | | | | | | | |
| --- | --- | --- | --- | --- | --- | --- | --- | --- | --- | --- | --- | --- |
| Item | Bedtime Resistance,Mean ± SD/ Median(IQR) | | *P* | Sleep Anxiety, Mean ± SD/ Median(IQR) | | *P* | Sleep Onset Delay,Mean ± SD/ Median(IQR) | | *P* | Daytime sleepiness,Mean ± SD/ Median(IQR) | | *P* |
|  | (-) | (+) |  | (-) | (+) |  | (-) | (+) |  | (-) | (+) |  |
| ABC |  |  |  |  |  |  |  |  |  |  |  |  |
| Sensory stimuli | 7(3-11) | 9(3-13) | 0.190 | 7(3-11) | 9(6-13) | ＜0.001 | 8(3-11) | 8(3.5-13) | 0.007 | 7(3-11) | 9(6-13) | 0.002 |
| Sensorial relating | 13(7-18) | 14(8-18.5) | 0.835 | 13(7-18) | 14.5(10-20) | 0.004 | 12.5±7.7 | 15.26±7.99 | ＜0.001 | 13(7-18) | 15(9.5-20.5) | 0.003 |
| Body and object use | 6(2-12) | 9(4-12.5) | 0.379 | 6(2-12) | 8(4-13.5) | 0.001 | 6(2.5-11) | 10(3-13.5) | ＜0.001 | 7(3-12) | 7(3-13) | 0.479 |
| Language | 12(6-17) | 11(6-16) | 0.020 | 11(6-17) | 12.5(7-19.75) | 0.223 | 11(6-17) | 13(6-19) | 0.015 | 11(6-17) | 14(7-19.5) | 0.125 |
| Social self-help | 10.71±5.28 | 10.55±5.11 | 0.676 | 11(7-14) | 12(9-16) | 0.004 | 10.39±5.25 | 12.01±4.92 | ＜0.001 | 10.52±5.25 | 11.49±5.06 | 0.045 |
| Total score | 49(35-66) | 53(38-67.5) | 0.729 | 48(35-64) | 57(40-76) | 0.001 | 49(35-65) | 56(41.5-78) | ＜0.001 | 49(35-65) | 60(41-75) | 0.003 |
| SRS |  |  |  |  |  |  |  |  |  |  |  |  |
| Social awareness | 11(9-13) | 13(11-15) | 0.009 | 11(9-13) | 13(11-15) | ＜0.001 | 11(9-13) | 13(11-15) | ＜0.001 | 11(9-13) | 13(10-15) | ＜0.001 |
| Social cognition | 17.83±4.84 | 18.56±4.23 | 0.031 | 17.65±4.76 | 19.22±4.37 | ＜0.001 | 18(15-21) | 18(16-23) | 0.004 | 18(15-21) | 20(16-23.5) | ＜0.001 |
| Social communication | 31.82±9.13 | 33.51±9.25 | 0.017 | 31.54±9.14 | 34.56±8.92 | ＜0.001 | 31.63±9.1 | 34.98±9.04 | ＜0.001 | 31.62±9.17 | 35.55±8.48 | ＜0.001 |
| Social motivation | 14(12-18) | 14(11-19.5) | 0.526 | 14(11-18) | 16(12.25-21) | ＜0.001 | 14(11-18) | 15(12-20) | ＜0.001 | 14(11-18) | 16(12.5-19) | 0.002 |
| Autistic mannerisms | 13(9-17.5) | 14(9-18) | 0.733 | 13(9-17) | 16(11-20.75) | ＜0.001 | 13(9-17) | 16(11-20) | ＜0.001 | 13(9-17.5) | 16(12-19) | ＜0.001 |
| Total score | 89.09±23.76 | 92.58±23.36 | 0.056 | 87.84±23.26 | 97.13±23.85 | ＜0.001 | 89(74-105) | 99(81-118) | ＜0.001 | 88.31±23.57 | 98.97±22.41 | ＜0.001 |
| CARS | 33(30-39) | 31(27.5-36) | 0.002 | 33(29-38) | 34(28.25-37.75) | 0.502 | 33(29-38) | 33(29-38.25) | 0.234 | 33(29-37.75) | 35(30-41) | 0.002 |
| Communication warning behavior | 43(26-59) | 40(29.5-60) | 0.951 | 42(26-58) | 45(30-62) | 0.104 | 42(27-58) | 43(28-68.5) | 0.061 | 42(27-59) | 47(30-64) | 0.047 |
| * There were 1,077 boys with ASD | | | | | | |  |  |  |  |  |  |
