# Supplementary material for: Sleep problems in children with autism spectrum disorder: a multicenter survey
Source: BMC Psychiatry. 2021 Aug 16;21:406. doi: 10.1186/s12888-021-03405-w (PMC8365936; doi:10.1186/s12888-021-03405-w)
Supplement: Supplementary file 4 — Additional file 4: Table S4. Differences in autism syptoms in ASD girls* with and without common sleep problems. [file 12888_2021_3405_MOESM4_ESM.docx]

| **Table S4.** Differences in autism syptoms in ASD girls* with and without common sleep problems | | | | | | | | | | | | |
| --- | --- | --- | --- | --- | --- | --- | --- | --- | --- | --- | --- | --- |
| Item | Bedtime Resistance,Mean ± SD/ Median(IQR) | | *P* | Sleep Anxiety, Mean ± SD/ Median(IQR) | | *P* | Sleep Onset Delay,Mean ± SD/ Median(IQR) | | *P* | Daytime sleepiness,Mean ± SD/ Median(IQR) | | *P* |
|  | (-) | (+) |  | (-) | (+) |  | (-) | (+) |  | (-) | (+) |  |
| ABC |  |  |  |  |  |  |  |  |  |  |  |  |
| Sensory stimuli | 6(3-9.75) | 8.5(3-13.25) | 0.289 | 6(3-10) | 8(6-11.75) | 0.398 | 6(3-10) | 8(6-13.5) | 0.009 | 6(3-10) | 8(5-12.5) | 0.006 |
| Sensorial relating | 12.85±7.22 | 12.18±8.17 | 0.557 | 12.81±7.56 | 12.12±7.42 | 0.569 | 12(7-18.25) | 14.5(10.25-20.75) | 0.011 | 12(6.5-16.5) | 15(10-20.5) | ＜0.001 |
| Body and object use | 6.5(2-12) | 6.5(3-13.25) | 0.855 | 6(2-13) | 7(4-12.75) | 0.604 | 6.5(2-12.25) | 6.5(2.25-13) | 0.032 | 7(2-12) | 6(2.5-14) | 0.120 |
| Language | 11(5-16) | 13(8-18.25) | 0.273 | 11(4.75-16.25) | 13.5(8.25-17.75) | 0.670 | 11.5(4.75-17) | 12(6.25-15.75) | 0.957 | 10.86±7.17 | 12.98±8.56 | 0.154 |
| Social self-help | 10.89±4.99 | 10.62±5.33 | 0.720 | 9(6-14) | 12(10-14.75) | 0.665 | 10.7±5.03 | 11.21±5.32 | 0.558 | 10.43±5.16 | 12.38±4.45 | 0.029 |
| Total score | 49.89±21.53 | 50.61±27.1 | 0.833 | 47.5(32.75-62.25) | 54.5(43.25-71.5) | 0.484 | 46(32.75-63.25) | 52(41.5-65.75) | 0.016 | 46(32-63.5) | 53(36.5-63.5) | 0.011 |
| SRS |  |  |  |  |  |  |  |  |  |  |  |  |
| Social awareness | 11.46±3.29 | 12.07±3.47 | 0.249 | 11.42±3.43 | 12.31±2.96 | 0.104 | 11.35±3.39 | 12.79±2.87 | 0.015 | 11.43±3.51 | 12.41±2.43 | 0.039 |
| Social cognition | 17.99±4.78 | 18.58±4.41 | 0.427 | 17.87±4.47 | 19.06±5.23 | 0.123 | 18.02±4.88 | 18.72±3.73 | 0.403 | 18.01±4.75 | 18.73±4.38 | 0.377 |
| Social communication | 32.46±9.26 | 34.58±9.05 | 0.145 | 32.37±8.9 | 35.17±10 | 0.067 | 32.53±9.3 | 35.13±8.71 | 0.115 | 32.55±9.43 | 34.95±8.23 | 0.137 |
| Social motivation | 15.57±5.13 | 15.93±5.2 | 0.662 | 15.62±5.11 | 15.83±5.26 | 0.800 | 15.36±5.3 | 16.95±4.2 | 0.083 | 15.14±5.02 | 17.76±5.1 | 0.003 |
| Autistic mannerisms | 13.17±5.88 | 13.27±6.11 | 0.913 | 12.76±5.71 | 14.6±6.46 | 0.060 | 12(8-16) | 14(9.75-17) | 0.135 | 12(8-16) | 14(9.5-17) | 0.021 |
| Total score | 90.65±23.72 | 94.44±23.65 | 0.314 | 90.03±22.77 | 96.98±26.02 | 0.076 | 90.18±24.21 | 97.95±20.55 | 0.066 | 89.86±23.78 | 98.85±22.24 | 0.030 |
| CARS | 32.5(27.125-38) | 31.25(26.75-36.125) | 0.453 | 32(27-37) | 34(28.125-36.875) | 0.699 | 32(26.75-36.625) | 35(27.5-38.5) | 0.112 | 32(27.25-37) | 32(27-37.5) | 0.278 |
| Communication warning behavior | 41.5(17-60.75) | 51.5(38.5-59) | 0.679 | 41.5(17-56.5) | 57(46-65) | 0.059 | 47(18.75-61.25) | 47.5(24-52) | 0.968 | 50(18.5-59) | 40(25-64) | 0.823 |
| * There were 233 girls with ASD | |  |  |  |  |  |  |  |  |  |  |  |
