# Supplementary material for: Sleep problems in children with autism spectrum disorder: a multicenter survey
Source: BMC Psychiatry. 2021 Aug 16;21:406. doi: 10.1186/s12888-021-03405-w (PMC8365936; doi:10.1186/s12888-021-03405-w)
Supplement: Supplementary file 5 — Additional file 5: Table S5. Differences in developmental quotient in ASD boys with and without common sleep problems. [file 12888_2021_3405_MOESM5_ESM.docx]

| **Table S5.** Differences in developmental quotient in ASD children with and without common sleep problems | | | | | | | | | | | | |
| --- | --- | --- | --- | --- | --- | --- | --- | --- | --- | --- | --- | --- |
| Item | Bedtime Resistance,Mean ± SD/ Median(IQR) | | *P* | Sleep Onset Delay,Mean ± SD/ Median(IQR) | | *P* | Sleep Anxiety, Mean ± SD/ Median(IQR) | | *P* | Daytime sleepiness, Mean ± SD/ Median(IQR) | | *P* |
|  | (-) | (+) |  | (-) | (+) |  | (-) | (+) |  | (-) | (+) |  |
| CNBS-R2016 |  |  |  |  |  |  |  |  |  |  |  |  |
| Gross motor | 73(61-88) | 76(62-89.5) | 0.642 | 73(61.5-88) | 76(61-89.5) | 0.407 | 73.5(62-88) | 73(61-89) | 0.501 | 73(62-88) | 73(57-85.5) | 0.191 |
| Fine motor | 57(45-69) | 57(44-70.5) | 0.583 | 57(44-69) | 57(48.5-70.5) | 0.863 | 57(44-69) | 57(47-71.25) | 0.446 | 58(45-70) | 53(42-68) | 0.837 |
| Adaptive behavior | 60(47-73) | 60(45.5-70) | 0.791 | 59(46-72) | 61(47.5-71.5) | 0.77 | 60(46-72) | 58(46.25-69.75) | 0.651 | 60(47-72) | 58(44-70) | 0.163 |
| Language | 48(33-65.5) | 46(31.5-69.5) | 0.897 | 48(32-68) | 46(35-62) | 0.126 | 46(32-67) | 50.5(36-68.5) | 0.353 | 48(33-68.5) | 45(31.5-57.5) | 0.646 |
| Personal-social | 51(40-63.5) | 50(39-61.5) | 0.318 | 51(39-64) | 50(42.5-60) | 0.201 | 51(40-63) | 51(41.25-62) | 0.524 | 51(41-63) | 51(37.5-61) | 0.354 |
| GQ | 59(48-69) | 57(48-68) | 0.85 | 59(48-69) | 59(49-68) | 0.564 | 59(47.75-69) | 59.5(51-67.75) | 0.558 | 59(48-69) | 56(45.5-66) | 0.285 |
