# Supplementary material for: Sleep problems in children with autism spectrum disorder: a multicenter survey
Source: BMC Psychiatry. 2021 Aug 16;21:406. doi: 10.1186/s12888-021-03405-w (PMC8365936; doi:10.1186/s12888-021-03405-w)
Supplement: Supplementary file 6 — Additional file 6: Table S6. Differences in developmental quotient in ASD girls with and without common sleep problems. [file 12888_2021_3405_MOESM6_ESM.docx]

| **Table S6.** Differences in developmental quotient in ASD children with and without common sleep problems | | | | | | | | | | | | |
| --- | --- | --- | --- | --- | --- | --- | --- | --- | --- | --- | --- | --- |
| Item | Bedtime Resistance,Mean ± SD/ Median(IQR) | | *P* | Sleep Onset Delay,Mean ± SD/ Median(IQR) | | *P* | Sleep Anxiety, Mean ± SD/ Median(IQR) | | *P* | Daytime sleepiness, Mean ± SD/ Median(IQR) | | *P* |
|  | (-) | (+) |  | (-) | (+) |  | (-) | (+) |  | (-) | (+) |  |
| CNBS-R2016 |  |  |  |  |  |  |  |  |  |  |  |  |
| Gross motor | 79.94±23.44 | 75.33±19.23 | 0.227 | 77.4±23.42 | 82.86±17.23 | 0.200 | 76.5(59-87.25) | 71(61.75-80.75) | 0.783 | 71(59.5-85) | 77(62-88.5) | 0.357 |
| Fine motor | 61.03±20.68 | 56.22±17.65 | 0.157 | 58.81±19.94 | 62.51±19.74 | 0.330 | 59.57±21.42 | 59.69±14.82 | 0.967 | 58.78±20.29 | 62.41±18.48 | 0.331 |
| Adaptive behavior | 63.09±23.1 | 57.12±22.5 | 0.129 | 59.48±23.01 | 68.03±22.09 | 0.051 | 62.16±23.38 | 58.81±22.01 | 0.417 | 60.13±23.51 | 65.32±21.03 | 0.229 |
| Language | 47(31.25-65.25) | 37.5(28-52.25) | 0.017 | 44(28-58.25) | 50(37.25-72.75) | 0.506 | 47.5(28-66.5) | 41.5(31.25-49.25) | 0.388 | 43(28-57) | 50(30.5-70.5) | 0.151 |
| Personal-social | 57.88±21.62 | 52.9±19.35 | 0.166 | 55.92±21.61 | 58.11±18.93 | 0.586 | 56.24±21.74 | 56.83±19.05 | 0.875 | 56.06±21.3 | 57.54±20.33 | 0.707 |
| GQ | 63.39±20.01 | 57.08±16.68 | 0.054 | 60.49±19.48 | 65.26±18.13 | 0.194 | 58.5(45.75-69.25) | 49(37-61.5) | 0.834 | 60.6±19.33 | 64.62±18.87 | 0.264 |
